# Contrasting genetic variation and positive selection followed the divergence of NBS-encoding genes in Asian and European pears

**DOI:** 10.1186/s12864-020-07226-1

**Published:** 2020-11-19

**Authors:** Manyi Sun, Mingyue Zhang, Jugpreet Singh, Bobo Song, Zikai Tang, Yueyuan Liu, Runze Wang, Mengfan Qin, Jiaming Li, Awais Khan, Jun Wu

**Affiliations:** 1 College of Horticulture, State Key Laboratory of Crop Genetics and Germplasm Enhancement, Nanjing Agricultural University, Nanjing, 210095 Jiangsu China; 2 Plant Pathology & Plant-Microbe Biology Section, Cornell University, Geneva, NY 14456 USA

**Keywords:** NBS, Pear, Expansion, Positive selection, Nucleotide diversity

## Abstract

**Background:**

The NBS disease-related gene family coordinates the inherent immune system in plants in response to pathogen infections. Previous studies have identified NBS-encoding genes in *Pyrus bretschneideri* (‘Dangshansuli’, an Asian pear) and *Pyrus communis* (‘Bartlett’, a European pear) genomes, but the patterns of genetic variation and selection pressure on these genes during pear domestication have remained unsolved.

**Results:**

In this study, 338 and 412 NBS-encoding genes were identified from Asian and European pear genomes. This difference between the two pear species was the result of proximal duplications. About 15.79% orthologous gene pairs had Ka/Ks ratio more than one, indicating two pear species undergo strong positive selection after the divergence of Asian and European pear. We identified 21 and 15 NBS-encoding genes under fire blight and black spot disease-related QTL, respectively, suggesting their importance in disease resistance. Domestication caused decreased nucleotide diversity across NBS genes in Asian cultivars (cultivated 6.23E-03; wild 6.47E-03), but opposite trend (cultivated 6.48E-03; wild 5.91E-03) appeared in European pears. Many NBS-encoding coding regions showed Ka/Ks ratio of greater than 1, indicating the role of positive selection in shaping diversity of NBS-encoding genes in pear. Furthermore, we detected 295 and 122 significantly different SNPs between wild and domesticated accessions in Asian and European pear populations. Two NBS genes (*Pbr025269.1* and *Pbr019876.1*) with significantly different SNPs showed >5x upregulation between wild and cultivated pear accessions, and > 2x upregulation in *Pyrus calleryana* after inoculation with *Alternaria alternata*. We propose that positively selected and significantly different SNPs of an NBS-encoding gene (*Pbr025269.1*) regulate gene expression differences in the wild and cultivated groups, which may affect resistance in pear against *A. alternata*.

**Conclusion:**

Proximal duplication mainly led to the different number of NBS-encoding genes in *P. bretschneideri* and *P. communis* genomes. The patterns of genetic diversity and positive selection pressure differed between Asian and European pear populations, most likely due to their independent domestication events. This analysis helps us understand the evolution, diversity, and selection pressure in the NBS-encoding gene family in Asian and European populations, and provides opportunities to study mechanisms of disease resistance in pear.

**Supplementary Information:**

The online version contains supplementary material available at 10.1186/s12864-020-07226-1.

## Background

Pear (*Pyrus*), the third most important fruit tree species in the world, has been cultivated for more than 3000 years and is one of the oldest fruit trees in the world [1]. Due to independent domestication under distinct geographical conditions, Asian and European pears display prominent differences in genetic and phenotypic diversity [2, 3]. The wild populations of these two pear groups have likely experienced unique disease pressures due to their completely different habitats. For example, brown spot (caused by fungus *Stemphylium vesicarium*) and fire blight (caused by bacteria *Erwinia amylovora*) diseases lead to death of pear trees and threaten the pear industry in Europe and west Asia [4–10]. Black spot (caused by fungus *A. alternata*), scab (caused by fungus *Venturia nashicola*), ring rot (caused by fungus *Botryosphaeria berengeriana*), and bitter rot (caused by bacteria *Colletotrichum fructicola*) diseases, in turn, cause huge losses to pear production in China and east Asia [11–14]. Furthermore, independent domestications of Asian and European pear [3] might have intensified the selection on a few genomic regions that have relevance for the specific diseases associated with their cultivation habitats. Hence, characterizing disease-related gene families can provide opportunities to understand the role of divergence and domestication in shaping host resistance responses in pear.

Studying disease resistance mechanisms in wild and cultivated germplasm remains a major focus of pear breeding and improvement [13, 15–20]. For example, Pierantoni L et al. [15] identified two major resistance QTL against scab disease caused by *Venturia pirina* in European pear (*P. communis*), and the causative scab disease gene ‘*Vnk*’ was found in Asian pear [16]. Similarly, QTL have also been identified for resistance to fire blight and black spot disease [21] in *Pyrus ussuriensis* [17] and *P. communis* [18]. Interestingly, the QTL region for black spot disease resistance contained two NBS-encoding genes [13], which were up-regulated after infection with *A. alternata*, the pathogen causing the black spot disease in *P. calleryana* [20]. These results highlight the importance of studying disease-related gene families, their genetic diversity, and the selection pressure on them in both Asian and European pear. Other pear species also show different levels of disease resistance. For instance, *P. calleryana* is a wild species native to China with strong disease resistance that has been used for fire blight resistance in the US [20]. A genetic source of fire blight resistance was detected from *P. ussuriensis* and *P. pyrifolia*, which have been used to breed disease resistance pear cultivars like ‘Harrow Sweet’ and ‘Moonglow’ [22]. Furthermore, monogenic sources of scab resistance were detected in some Asian pear accessions including ‘Kinchaku’, ‘Cangxili’ and ‘Hongli’ [21]. These QTL, disease-resistance genes, and germplasm resources are potentially useful in understanding disease resistance mechanisms and breeding new disease-resistant cultivars in pear.

Plant disease resistance (R) genes play an important role in defense against pathogens and are classified based on their location in plant cells and their putative protein domain [23]. The nucleotide binding site plus leucine-rich repeat genes (NBS-LRR) represents the biggest class of R genes [24]. These genes mainly participate in the tracking and response of various pathogens including bacteria, viruses, fungi, nematodes and oomycetes [24, 25]. Recently, a range of NBS-encoding genes have been identified in different rosaceae species. For instance, the number of NBS-encoding genes varies from 346 to 1303 in strawberry and apple, respectively [26]. However, these numbers were different in other similar studies [27–29], probably due to different computational criteria used for their detection. It has been postulated that gene loss and expansion within species has caused the variation in number of NBS genes, and repeated duplication, divergence and eventual loss has facilitated their evolution in response to the rapidly changing pathogens [30]. In addition, NBS-encoding genes show a strong signature of selection, but the nature of selection differs across these genes [31]. Some NBS-encoding genes evolve rapidly through copy number variation and high ratio of non-synonymous to synonymous substitutions, but other genes evolve at a slower rate [31].

With the availability of new high-quality assembled genomes of double haploid *P. communis* [32] and *P. bretschneideri* [1], an accurate characterization of NBS-encoding genes and their comparative analysis with *P. bretschneideri* is feasible. In addition, whole-genome resequencing data from a large collection of pear accessions [3] also provides a massive genomic resource to study diversification and selection effects in pear. Since the selection and evolution of NBS-encoding genes is yet unclear, the genome sequence information provides chances to explore their diversity and evolution in wild and cultivated groups in Asian and European pear.

In this study, we identified NBS-encoding genes and compared them across the genomes of *P. bretschneideri* and *P. communis*. We further used re-sequencing data from 131 pear accessions to characterize genetic variation and selection signatures on NBS-encoding genes in wild and domesticated Asian and European pear populations. In addition, NBS-encoding genes in disease resistance QTL were identified in Asian and European pears. These findings will provide additional resources for future research of NBS-encoding gene function and disease resistance in pear.

## Result

### Identification and phylogenetic analysis of NBS-encoding genes in *P. bretschneideri* and *P. communis*

In this study, a total of 338 and 412 NBS-encoding genes were identified in the ~ 510 Mb *P. bretschneideri* (Asian pear cultivar: ‘Dangshansuli’) genome, and the ~ 497 Mb *P. communis* genome (European pear cultivar: ‘Bartlett’), respectively. The NBS-encoding genes were divided into six types including CC-NBS-LRR, TIR-NBS-LRR and four truncated NBS-LRR (Table 1), three of which (TIR-NBS-LRR, CC-NBS-LRR, NBS) determine the majority of the difference in the number of NBS-encoding genes between Asian and European pears. The NBS-LRR class was most frequent in both *P. bretschneideri* (36.4%) and *P. communis* (25.7%)*.* However, two different classes, CC-NBS-LRRs and NBS, represented the second largest classes in *P. bretschneideri* (26.6%) and *P. communis* (24.0%), respectively. The TIR-NBS (6.21%), CC-NBS (9.46%), NBS (10.36%), and TIR-NBS-LRR (10.95%) were least frequent in *P. bretschneideri*, while CC-NBS (7.04%), CC-NBS-LRR (9.22%), and TIR-NBS (13.35%) were smallest classes in *P. communis* (Table 1). The percentages of LRR domain-containing NBS genes were approximately 74.0 and 55.6% in *P. bretschneideri* and *P. communis*, respectively. However, the non-LRR containing genes showed a clear difference between Asian (*n* = 88; 26.04%) and European (*n* = 183; 44.41%) pear genomes (Table 1).

**Table 1:** Classification of NBS-encoding genes in ‘Dangshansuli’ and ‘Bartlett’

Type^a^	Numbers	
	‘Dangshansuli’	‘Bartlett’
CC -NBS-LRR	90	38
TIR- NBS- LRR	37	85
**Truncated NBS-LRR**^b^		
NBS-LRR	123	106
TIR- NBS	21I	55
CC-NBS	32	29
NBS	35	99
**Total NBS**	338	412
Total NBS with LRR	250	229
Total NBS without LRR	88	183

^a^Based on the presence or absence of CC, TIR, NBS and LRR domains, the NBS genes were classified into CC-NBS-LRR, TIR-NBS-LRR and four Truncated NBS-LRR types. ^b^Truncated NBS-LRR means NBS genes lacking N-terminal domain (TIR or CC) or C-terminal domain (LRR), and classified into NBS-LRR, TIR-NBS, CC-NBS, NBS

**Fig. 1:**
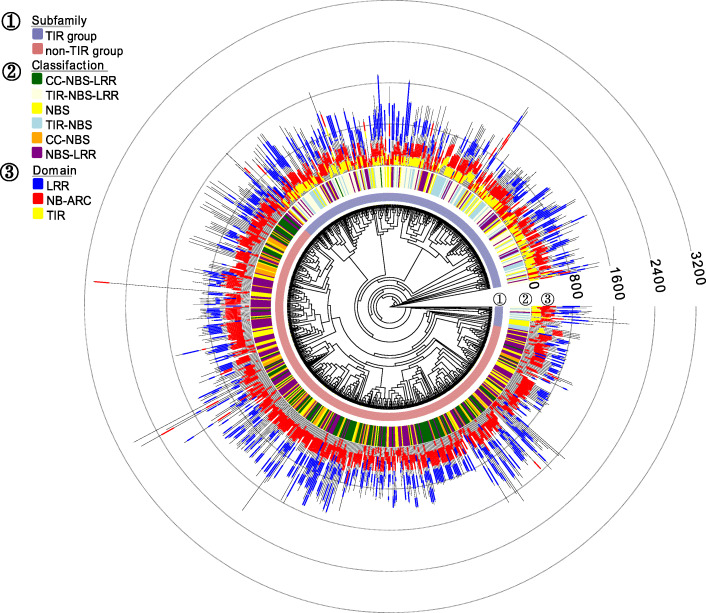
The maximum likelihood (ML) Phylogenetic analysis of NBS-encoding genes from *P. bretschneideri* and *P. communis*. The phylogenetic tree was obtained using the IQ-TREE software on the basis of amino-acid sequences of NBS-encoding genes with ultrafast bootstrap 1000. ① Two subfamilies are shown. Red represents non-TIR group and Blue represents TIR group. ② The six classes of NBS-encoding genes are marked by different colors. Green means CC-NBS-LRR type, light yellow means TIR-NBS-LRR type, yellow means NBS type, light blue means TIR-NBS type, orange means CC-NBS type and purple means NBS-LRR type. ③ Domains of NB-ARC, LRR and TIR are displayed on the tree (CC domain was not shown), yellow means TIR domain, red means NB-ARC domain and blue means LRR domain

Phylogenetic analysis revealed that most non-TIR-NBS type genes and TIR-NBS genes were divided into two clear groups (Fig. 1), and this phenomenon remained consistent in the separate analysis of *P. bretschneideri* and *P. communis* (Additional file 1, Figure S1, S2). This analysis further showed that most NB-ARC domains were located at similar positions on the genes, with lengths ranging from 250 to 300 bp. The position, number, and length of LRR domains were comparatively more variable in NBS-encoding genes. In addition, a large number of clades specific to *P. bretschneideri* and *P. communis* were displayed on the phylogenetic tree. Many species-specific genes and the differences in the numbers of NBS-encoding genes might have appeared after the divergence of Asian and European pears [3].

Promoter region (~ 1500 bp) analysis identified 128 different kinds of cis-elements in the NBS-encoding gene families of *P. bretschneideri* and *P. communis*, with 16 cis-elements having frequency more than 40% among them (Table 2). The CAAT-box, TATA-box, and MYC cis-elements showed highest enrichment in the promoters of NBS-encoding genes of both *P. bretschneideri* and *P. communis.* These 16 cis-elements were associated with response to various environmental factors (light, temperature, water and anaerobic stresses) and plant hormones (jasmonic acid, methyl ester, ethylene and salicylic acid). Interestingly, five types of cis-elements only appeared in *P. communis*; two of them were H-box and JERE, which regulate defense and jasmonate-responsive genes.

**Table 2:** Frequency of cis-elements identified in the promoter regions of NBS-encoding genes in two pear species

Cis-element name	Sequence	Putative response	Frequency^a^	
			‘Dangshansuli’	‘Bartlett’
CAAT-box	CCAAT	Seed	1.00	1.00
TATA-box	TATAA	core promoter element	1.00	1.00
MYC	CATTTG	Cold	0.95	0.96
ARE	AAACCA	anaerobium	0.75	0.74
STRE	AGGGG	Stress	0.72	0.73
ABRE	ACGTG	ABA	0.69	0.74
Box 4	ATTAAT	Light	0.69	0.72
CGTCA-motif	CGTCA	MeJA	0.67	0.75
TGACG-motif	TGACG	MeJA	0.67	0.75
TCT-motif	TCTTAC	Light	0.48	0.51
GT1-motif	GGTTAA	Light	0.49	0.46
MBS	CAACTG	Drought	0.49	0.52
ERE	ATTTCATA	Ethylene	0.46	0.49
W box	TTGACC	phytochrome down-regulation	0.43	0.45
LTR	CCGAAA	low-temperature	0.41	0.46
TCA-element	CCATCTTTTT	salicylic acid	0.39	0.49

^a^Frequency = Genes (containing this cis-element)/All NBS-encoding genes in Dangshansuli (Bartlett). Cis-elements on upstream 1500 bp sequence of NBS genes were identified by Plant CARE (http://bioinformatics.psb.ugent.be/webtools/plantcare/html/) program. Genome sequences of Dangshansuli and Bartlett were downloaded from NJAU (http://peargenome.njau.edu.cn/) and phytozome database (https://phytozome.jgi.doe.gov/)

### Genome-wide distribution and duplication of NBS-encoding genes

**Fig. 2:**
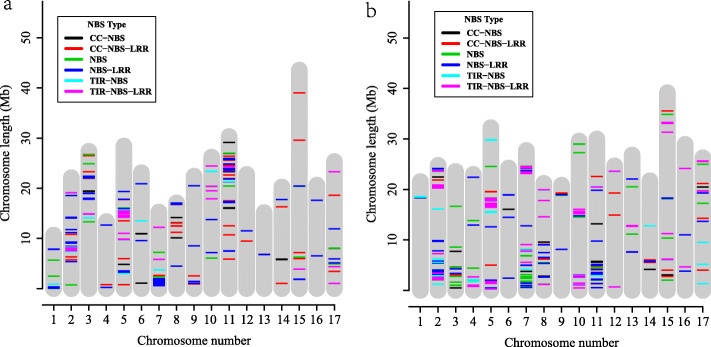
Chromosome location of NBS-encoding genes in *P. bretschneideri* (**a**) and *P. communis* (**b**). Different colors represent different types of NBS-encoding genes. Blue (NBS-LRR), purple (TIR-NBS-LRR), red (CC-NBS-LRR), green (NBS), cyan (TIR-NBS), black (CC-NBS). The x-axis represents the chromosome number, and the y-axis represents chromosome length

In *P. bretschneideri*, a total of 265 NBS-encoding genes were mapped to the 17 chromosomes and the remaining 73 genes were placed on scaffolds (Additional file 2). In the mapped genes, the highest number, 151 genes (56.98%), were located on four chromosomes; Chr2 (37), Chr5 (52), Chr7 (59), and Chr11 (50) (Fig. 2a). In *P. communis*, 408 NBS-encoding genes were mapped onto the 17 chromosomes, while 4 genes were on the unplaced scaffolds. As in *P. bretschneideri*, four chromosomes, Chr2 (52), Chr5 (42), Chr7 (58), Chr11 (41), had the highest proportion of the total NBS-encoding genes (Fig. 2b). A set of 40.38% (21/52) NBS-encoding genes on Chr2 co-localized with the previously detected fire blight disease resistance QTL of *P. communis* (Additional file 3) [13, 16, 17, 22, 33–35]. In addition, 30% (15/50) NBS-encoding genes on Chr11 were located within the black spot disease resistance QTL of *P. bretschneideri* (Additional file 3).

To detect the potential mechanism of expansion, we analyzed the duplication events of NBS-encoding genes in the *P. communis* and *P. bretschneideri* genomes. Each member of the NBS-encoding gene family was divided into five different categories: singleton, WGD or segmental, tandem, proximal, or dispersed. We found that 21,493 (50.20%) genes in the *P. bretschneideri* genome and 20,250 (54.08%) genes in the *P. communis* genome (data not shown) were primarily derived from WGD or segmental duplications. However, only 23.96% of NBS-encoding genes in *P. bretschneideri* and 19.90% in *P. communis* were duplicated and retained from WGD events. These results indicated that the percentage of NBS-encoding genes derived from dispersed and proximal duplication were 27.81 and 28.11% in *P. bretschneideri*, and 20.15 and 40.05% in *P. communis*, respectively (Additional file 4).

### Time of duplication events and selection pressure in NBS-encoding genes

**Fig. 3:**
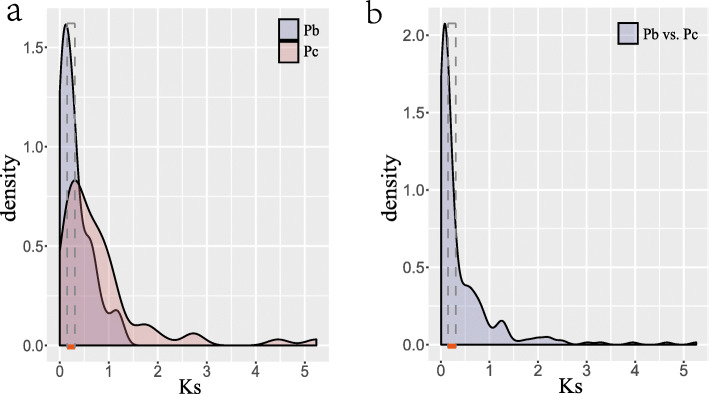
Distribution of Ks values of NBS genes pairs. Orange lines at the x-axis and the gray dotted box represented the Ks value region from 0.15 to 0.30. **a** Distribution of Ks values in *P. bretschneideri* and *P. communis*, respectively (**b**) Distribution of Ks values of Orthologous gene pairs between two pear species. Letter ‘Pb’ means *P. bretschneideri*, and letter ‘Pc’ means *P. communis*. Ks represents synonymous substitutions per site

By estimating evolutionary dates with the synonymous substitution rate per site (Ks), we identified that pear genome had undergone two WGD events [1]; an ancient WGD (Ks ~ 1.5–1.8) that occurred about 140 MYA ago [36], and a recent WGD (Ks ~ 0.15–0.3) event that may have occurred 30–45 MYA ago [1]. In *P. bretschneideri*, a total of 60 duplicated NBS-encoding gene pairs located on the collinear blocks were identified and, out of them, Ks values of 16 gene pairs (26.66%) ranged from 0.15–0.30 (Fig. 3a) [1]. As such, *P. communis* had 61 duplicated NBS-encoding gene pairs (Fig. 3a) and Ks values of 13 (21.31%) of these genes ranged from 0.15–0.30. The duplicated gene pairs derived from ancient WGD events have been lost in *P. bretschneideri*, but three duplicated gene pairs (Ks ~ 1.7) were retained in *P. communis*.

We further estimated the ratio of non-synonymous (Ka) and synonymous substitutions per site (Ks) to investigate the role of positive and purifying selection in the evolution of NBS-encoding genes. For a total of 60 duplicated gene pairs in *P. bretschneideri*, the Ka/Ks ratio of 3 pairs was more than 1; the Ka/Ks ratio of the other 57 gene pairs were less than 1 (Additional file 5). Likewise, out of total 61 duplicated pairs in *P. communis*, only 1 pair had Ka/Ks ratios > 1 while the remaining 60 pairs have Ka/Ks < 1.

**Fig. 4:**
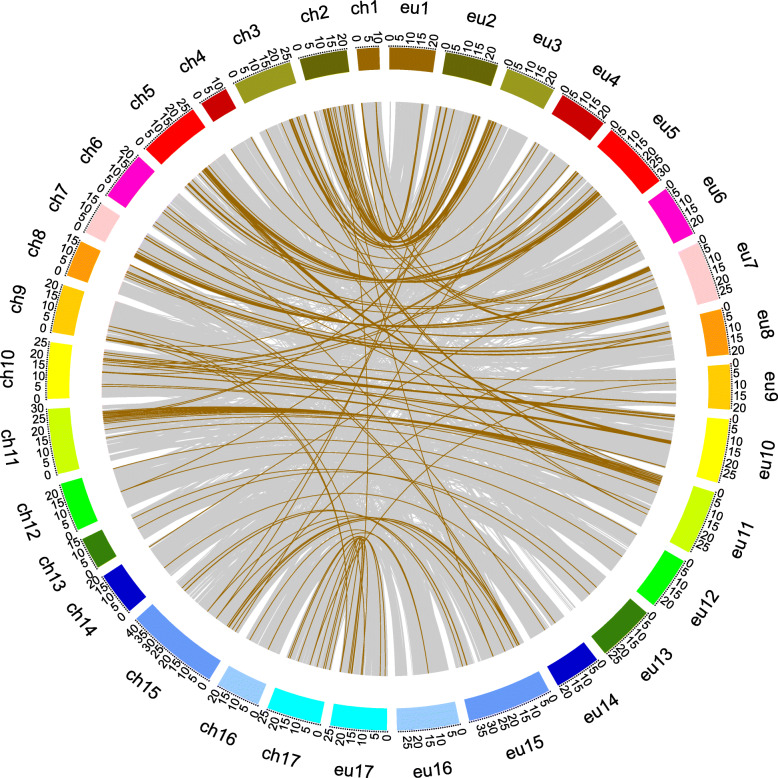
Synteny analysis of the NBS-encoding genes between *P. bretschneideri* and *P. communis*. Letter ‘ch’ means the chromosome of *P. bretschneideri* and letter ‘eu’ means the chromosome of *P. communis*. Brown lines means collinearity relationships among genes between *P. bretschneideri* and *P. communis*

A total of 266 orthologous gene pairs were identified between *P. bretschneideri* and *P. communis* (Additional file 6), and most orthologous gene pairs were present on the homologous chromosomes of two pear species (Fig. 4). Ks values of these gene pairs ranged from 0.006 to 5.25 (Fig. 3b). In addition, the Ka/Ks values of 266 orthologous gene pairs ranged from 0.15–2.51 (Additional file 7). A set of 42 orthologous gene pairs had Ka/Ks value > 1. These results indicated that about 35.21% NBS-encoding genes of *P. bretschneideri* had lost their orthologous genes in *P. communis,* and 17.79% orthologous gene pairs underwent positive selection.

### Genetic diversity and selection across NBS-encoding genes in Asian and European pear

An analysis of genetic diversity using genome sequences from 131 Asian and European pear accessions (including 61 wild and 70 cultivated accessions) revealed a total of 50,682 SNPs in NBS-encoding genomic regions, out of which 22,472 SNPs were present in the coding regions. After removing heterozygous SNPs, we obtained 7654 synonymous and 14,481 non-synonymous SNPs, and a non-synonymous/synonymous ratio of 1.89.

**Fig. 5:**
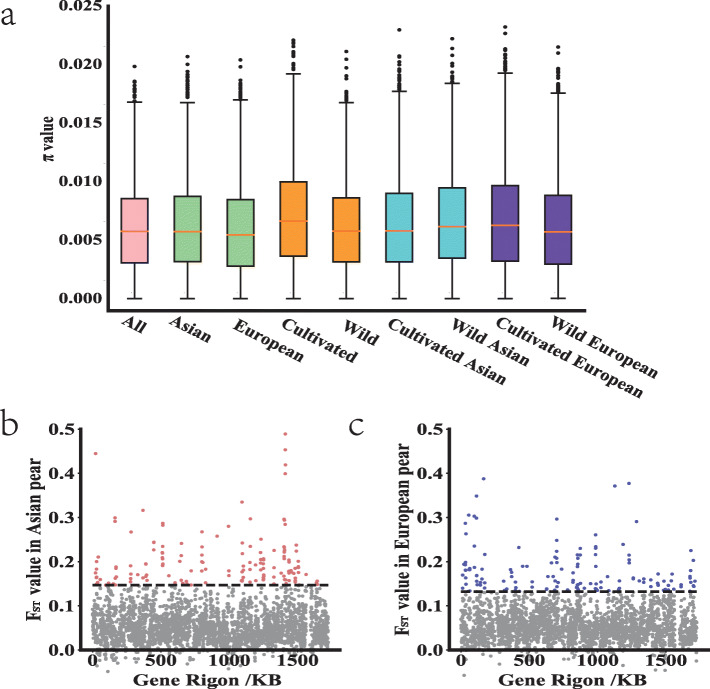
**a** Distribution of nucleotide diversities (*π*) in different pear groups. Pink (all pear accessions), green (Asian and European), orange (Cultivated and wild), blue (Asian cultivated and Asian wild), purple (European cultivated and European wild). **b** Distribution of *F*_*ST*_ values across the whole NBS-encoding gene family of Asian pears. Red plot is the top 5% *F*_*ST*_ value (> 0.14) in Asian pears. **c** Distribution of *F*_*ST*_ values across the whole NBS-encoding gene family of European pears Blue plot is the top 5% *F*_*ST*_ value (> 0.13) in European pears

The average nucleotide diversity across NBS-encoding genes was 6.02E-03 and 5.65E-03 in the Asian and European populations, and 5.92E-03 and 6.88E-03 in the wild and cultivated groups (Additional file 8), respectively. A similar trend was observed when analysis was repeated using only the coding regions from NBS-encoding genes (Table 3). In general, nucleotide diversity had a wide distribution ranging from 0.02 to 2.19E-05 in Asian and 0.01 to 2.48E-05 in the European pears, respectively (Fig. 5a; Additional file 8). A comparison of cultivated and wild populations revealed that average nucleotide diversity across NBS-encoding genes reduced after domestication in Asian pears, but contrasting results were observed in the European population (Table 3). However, individual NBS gene coding regions showed varied patterns of genetic diversity in wild and cultivated groups in both Asian and European pear populations. About 20% NBS gene coding regions showed a minimum 4-fold loss of nucleotide diversity in cultivated accessions than the wild germplasm in Asian pear (Additional file 8). Similarly, 17.2% of NBS-encoding regions had 4-fold genetic diversity loss during domestication in European pear. A total of 41 NBS-encoding genomic windows having reduction in genetic diversity were common in Asian and European populations (Additional file 8). Further analysis of fixation index across the whole NBS-encoding gene family revealed similar (T test, *p*-value = 0.31) average fixation index (*F*_*ST*_) values in Asian (5.77E-02; Fig. 5b) and European (5.89E-02; Fig. 5c) pear groups. However, similar to nucleotide diversity analysis, specific NBS gene coding regions showed high divergence between wild and cultivated groups in both the pear populations. For example, we found that 6 and 5 genes have *F*_*ST*_ values more than 0.30 in Asian and European populations, respectively.

**Table 3:** Nucleotide diversity (*π*) of NBS gene family in different pear groups

Pear accessions—groups (number of samples)	***π***	***π*** (Coding region)
All (131)	5.88E-03	4.75E-03
Wild (61)	5.92E-03	4.82E-03
Cultivated (70)	6.84E-03	5.46E-03
Asian (71)	6.02E-03	4.74E-03
European (60)	5.65E-03	4.76E-03
Asian wild (34)	6.47E-03	5.15E-03
Asian cultivated (37)	6.23E-03	4.88E-03
European wild (27)	5.91E-03	5.02E-03
European cultivated (33)	6.48E-03	5.40E-03

The nucleotide diversity was calculated by VCFtools with a 1000 bp sliding windows and step size of 500 bp. The sequence data of 131 pear accessions were obtained from previous study [3]

A chi-square analysis revealed that a greater number of significantly (*p*-value < 0.01) different SNPs appeared in Asian (295) than European (122) population (Table 4; Additional file 9), and the number of non-synonymous SNPs also followed a similar trend (184 vs. 74) (Table 4; Additional file 10). We further calculated the Ka/Ks ratio to investigate positive selection in the 338 NBS-encoding genes. A set of 64 and 60 genes had signatures of positive selection (Ka/Ks > 1) in Asian wild and cultivated groups (Table 4), and 51 genes were noticed in both groups (Additional file 11). A total of 57 and 63 genes had signatures of positive selection (Ka/Ks > 1) in European wild and cultivated group (Table 4), and 51 genes were in both groups. The trend in the number of genes under positive selection was very similar to the nucleotide diversity patterns in the four groups.

**Table 4:** Summary of genetic variation and genes with signature of positive selection in different populations

Population	Number of significantly different SNPs^**1**^	Number of non-synonymous SNPs^a^	Number of genes having non-synonymous SNPs	Subpopulation	Genes having signature of positive selection^b^
Asian	295	184	61	Wild	64
				Cultivated	60
European	122	74	39	Wild	57
				Cultivated	63

^1^Significantly different SNPs means this SNP has significant (chi-square test *p*-value < 0.01) different alleles distribution between wild and cultivated groups. ^a^Non-synonymous SNP means the significantly different SNP was also non-synonymous. ^b^Genes with Ka/Ks ratio more than one were considered as having signature of positive selection in different populations

### Expression analysis of genes in wild and cultivated Asian pear

**Fig. 6:**
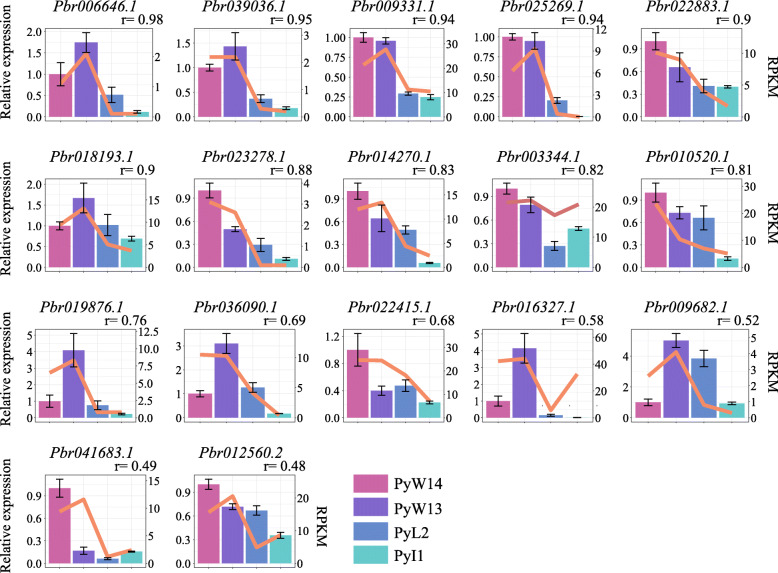
Expression profiles of 17 NBS-encoding genes in four pear accessions at enlarged stage by RT-qPCR analysis and RNA-seq data. The relative expression levels of each individual NBS-encoding genes were normalized by the *Pyrus* GAPDH gene. The expression level of PyW14 sample was used as a reference (relative expression level = 1). The letter “r” represents the correlation between RNA-seq data and RT-qPCR data. Bars represent mean relative expression level of genes (rose red: PyW14, purple: PyW13, blue: PyL2, cyan: PyI1); lines represent value of RPKM. Mean relative expression value and corresponding standard deviation were calculated by the three replicates of relative expression value. Four pears included Asian wild (PyW13,14) and cultivated (PyL2,PyI1) accessions. Left y-axis represents the relative expression level and right y-axis represents RPKM values

We investigated the expression of a total of 17 genes expression. 16 of 17 genes had more than 2-fold mean Reads Per Kilobase per Million mapped reads (RPKM) difference between two wild pears and two cultivated pears, and another gene (*Pbr003344.1*) was in black spot disease QTL (Additional file 12). These genes, *Pbr019876.1*, *Pbr039036.1* and *Pbr025269.1*, had significantly (*p*-value < 0.01) different SNPs between Asian wild and cultivated pear accessions and showed > 3-fold higher expression in 2 wild accessions than 2 cultivated pears at three different fruit developmental stages from RNA-Seq analysis (Fig. 6).

A quantitative real-time PCR (RT-qPCR) analysis of 17 genes also displayed consistent expression patterns as noticed in the RNA-seq analysis (Fig. 6). To investigate the role of these 17 genes in pathogen response, we compared RNA-seq data of control and *A. alternata* inoculated groups in accessions of a wild pear (*P. calleryana*) [20] and a cultivar of ‘Hongfen’ pear (*P. pyrifolia*) [19] (Additional file 13). Genes *Pbr019876.1*, *Pbr025269.1*and *Pbr018193.1* had upregulated (> 2-fold) expression in the inoculated than control samples of *P. calleryana*. The NBS-encoding gene, *Pbr003344.1*, under black spot disease resistance QTL also had up-regulated (~ 1.9-fold) expression after inoculation of *A. alternata* in *P. calleryana*.

### A model to identify putative disease resistance in pear

**Fig. 7:**
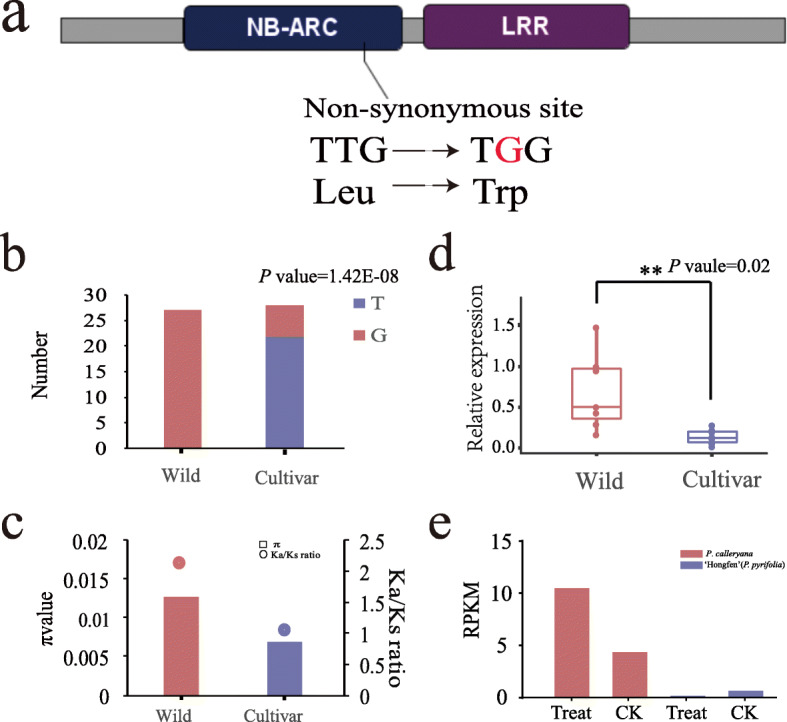
Divergence of *Pbr025269.1* between Asian cultivated and wild groups. **a** One significantly different non-synonymous SNP located on NBS domain. **b** Genotyping distribution of SNP1 in Asian cultivated and wild groups (chi-square test, *p*-value = 1.42E-08). **c** Ka/Ks ratio and nucleotide diversity of *Pbr025269.1* in Asian wild and cultivated groups. Bars mean nucleotide diversities and spots mean Ka/Ks ratios. **d** Expression profiles of *Pbr025269.1* in ten Asian cultivated (PyL2, PyL7, PyL8, PyL9, PyL10, PyL11, PyI1, PyI9, PyI11, PyI14) and seven Asian wild (PyW5, PyW6, PyW7, PyW9, PyW12, PyW13, PyW14) pear accessions at enlarged stage by RT-qPCR analysis. The relative expression levels of each individual NBS-encoding genes were normalized by the *Pyrus* GAPDH gene. The expression level of PyW14 sample was used as a reference (relative expression level = 1). Each point means relative expression level of one pear accession. ‘**’ means significant difference (T-test, *p*-value< 0.05) between wild and cultivated groups. (e) RPKM value of *Pbr025269.1* in *P. calleryana* and ‘hongfen’ pear (an Asian cultivate pear), ‘Treat’ means inoculated *A. alternata* groups and ‘CK’ means control groups; red represents *P. calleryana*, blue represents ‘hongfen’(*P. pyrifolia*)

In order to detect the putative formation of resistance in wild and cultivated pear accessions, we combined the analysis of genetic variants, diversity, selection pressure and expression patterns in NBS-encoding genes. It was found that NBS-encoding gene of *Pbr025269.1* had 36 significantly (chi-square test *p*-value < 0.01) different SNPs between Asian wild and cultivated groups (Fig. 7a, b), and one of the SNP mutation caused amino acid change from leucine to tryptophan (Fig. 7a). This gene had higher nucleotide diversity in wild (1.22E-02) than cultivated (6.36E-03) group (Fig. 7c), had high divergence (*F*_*ST*_ *=* 0.32*)* between Asian wild and cultivated groups, and showed a stronger signature of positive selection (Ka/Ks ratio = 2.04) in wild than cultivated (Ka/Ks ratio = 1.06) Asian pear accessions (Fig. 7c). Furthermore, *Pbr025269.1* also showed significantly (*P* value =0.02) different expression between wild and cultivated accessions in Asian pears (Fig. 7d). Expression level of *Pbr025269.1* in the *A. alternata* inoculated *P. calleryana* (an Asian wild pear) accession was higher than observed in control groups (Fig. 7e), but this up-regulation did not appear in ‘Hongfen’ pear (Asian cultivated pear). Overall, positively selected *Pbr025269.1* had significantly different SNPs and gene expression between the wild and domesticated Asian pear accessions.

## Discussion

### Difference in genetic variation patterns across the NBS-encoding genes between Asian and European pears

The NBS-encoding gene family has the most ancient and prevalent disease resistance genes, which play an important role in protecting plants from infection by diverse pathogens [37]. In this study, we observed a similarity in the distribution patterns and cis-elements types of NBS-encoding genes in *P. communis* and *P. bretschneideri*, indicating the conservation of core NBS genes before the divergence of these two pear species. The number of NBS-encoding genes in *P. bretschneideri* and *P. communis* were considerably different (338 vs 412), and only 64.79% orthologous NBS-encoding genes were present between them. Both Asian and European pear genomes had almost similar genome content, which did not define the differences in the number of NBS genes observed in this study [1, 32]. These differences might have originated from gene duplication and gene loss events associated with their adaptation to varying environments [38–40] during diversification of Asian and European pears approximately 6.6 and 3.3 MYA [1]. Due to the high heterozygosity and highly repetitive genome (53.1% repetitive sequences) of pear, 73 (21.60%) NBS genes of ‘Dangshansuli’ pear were not mapped to the chromosomes [1]. In addition to the gene gain/loss events, we also noticed higher nucleotide diversity in the cultivated pear group than the wild group from Europe (6.48E-03 vs. 5.91E-03), but the trend was opposite in the Asian population (wild vs. cultivated, 6.47E-03 vs. 6.23E-03). These findings suggest that a domestication-related loss of genetic diversity happened in Asian pear group. In contrast, the higher nucleotide diversity in NBS-encoding genes of cultivated groups in Europe might have occurred due to distinct selection pressure imposed by the cultivated habitats [41]. A recent study has shown that several regions with NBS-encoding genes had higher nucleotide diversity in landraces than the wild pear group [42]. The latter results, along with our findings, indicate that the genetic diversity of similar genes can be altered differently due to preferential selection of traits during independent domestication events as observed in pear [3]. This proposition was further supported by the identification of distinct NBS genes in Asian and European pears that showed reduction in genetic diversity after domestication bottleneck. Overall, the characterization and genetic diversity analysis suggest that NBS-encoding genes have undergone distinct selection pressures in pear due to its independent domestication in Asia and Europe.

The level of differentiation of NBS-encoding genes between wild and cultivated groups between two pear species also differs significantly from some other annual species. For instance, only 6 and 5 NBS-encoding genes show high divergence (*F*_*ST*_ value > 0.30) between wild and cultivated groups from Asia and Europe, respectively, whereas 17 genes had high *F*_*ST*_ values (> 0.30) between *Indica* and *nivara* rice species [43]. These differences reflect a much longer domestication history (> 10,000 years) of rice [43] than the cultivated pear (~ 3300 years) [44]. The longer generation time and perennial nature might have also led to few sexual generations during pear domestication [3], which is a key factor causing lower divergence of NBS-encoding genes in cultivated and wild pear groups than annual crops.

### Positive selection drives the evolution and selection of NBS-encoding genes in Asian and European pears

Ka/Ks ratio of several paralogous gene pairs were greater than one, indicating a positive selection on NBS-encoding gene family in pear. The NBS-encoding regions also had higher nonsynonymous / synonymous ratio (1.89) than the genome averages (1.20), a consistent pattern also seen in rice [41]. Moreover, the number of gene pairs showing signs of positive selection differed between *P. communis* and *P. bretschneideri,* with the latter showing comparatively stronger selection and more rapid evolution of NBS-encoding genes. The NBS-encoding genes also exhibited positive selection and rapid evolution in other species [26, 45]. Positive selection facilitates the prevalence of advantageous traits for the evolution of particular species [46–48], hence the signatures of positive selection indicate the increased fitness of NBS-encoding genes to overcome disease pressure by accumulating favorable alleles during evolution. These results also suggest that *P. communis* and *P. bretschneideri*, endured different disease pressures during their divergence and domestication. For example, fire blight and brown spot damage was reported in pear production in Europe and west Asia [4–10], while ring rot, bitter rot, and scab infections occurred in China and east Asia [11–14]. The co-evolution with local pathogen populations and distinct disease pressure on pear production in different geographical areas can explain the difference in selection patterns across NBS-encoding genes in Asia and Europe.

We also found that the high percentage of these positively selected genes appeared in both wild and cultivated groups, indicating that these genes may play important roles in both wild and cultivated populations. Furthermore, a high percentage of non-synonymous SNPs showed significant (chi-square test *p*-value < 0.01) differences in Asian and European populations. These results were consistent with an existing study showing that significant divergent non-synonymous SNPs appeared among *M. x domestica* and other wild groups [49]. Host plants accumulate non-synonymous mutations to confer resistance against evolving pathogens [50], and higher frequency of these significantly different mutations in Asian and European pears suggest distinct pathogen pressure accompanying their independent domestication [37].

### NBS-encoding genes and disease resistance in pear

The presence of 41 NBS-encoding genes in the previously identified disease-related QTL regions indicated their important roles in defense against these diseases. For example, two major QTL have been identified on chromosome 2 for fire blight resistance in ‘Moonglow’ (*P. communis*) and ‘Harrow Sweet’ (*P. communis*) pear cultivars [22, 34]. The distribution analysis has identified NBS gene clusters in these two QTL regions. Similarly, 15 NBS-encoding genes were located under the black spot resistance QTL on chromosome 11 of *P. bretschneideri*. Three NBS-encoding genes (*Pbr023226.1, Pbr023227.1, Pbr003344.1*) in the black spot QTL regions showed > 1.9-fold expression after *A. alternata* inoculation in *P. calleryana* [20]. These gene expression differences were not significant after *A. alternata* inoculation in a susceptible ‘Hongfen’ pear (*P. pyrifolia*) [19], suggesting the specificity of gene expression based on the cultivar genetic background*.* Nonetheless, these expression differences might reflect the disease susceptibility or resistance depending on the genetic background of pear genotypes. Furthermore, the QTL-spanning NBS-encoding genes on chromosome 2 and 11 may serve as important functional candidates to study the genetic mechanisms of disease resistance against fire blight, pear scab and black spot in pear. For example, a detailed analysis identified positive selection and a high differential expression level of *Pbr025269.1*, a homolog of *AT3G07040* to *Pseudomonas syringae pv. Maculicola* resistance [51, 52], in wild pears. This gene controls cell death at the site of infection [53, 54]. Transcriptome analysis also showed difference in expression of *Pbr025269.1* between *P. calleryana* and ‘Hongfen’ pear (*P. pyrifolia*) after infection with the *A. alternata* pathogen [55]. Thus, *Pbr025269.1*-like gene candidates can serve as functional candidates to fine map a QTL-associated resistance and identifying their role in pathogen defense.

## Conclusion

The NBS-encoding gene family evolved through duplication events in *P. bretschneideri* and *P. communis*, and proximal duplication has resulted in different numbers of NBS-encoding genes in these two pear species. At a population level, the patterns of genetic diversity and positive selection were different across Asian and European pears. Moreover, several NBS-encoding genes were located under disease resistance QTL regions, indicating their potential role in disease resistance or susceptibility in pear. One specific NBS-encoding gene, *Pbr025269.1*, has shown signatures of selection, and exhibits high expression levels after inoculation with *A. alternata*.

## Methods

The pear genome was downloaded from Pear Center of Nanjing Agricultural University. The genome resequencing data from 113 pear accessions were obtained from previous study [3], and additional resequencing data from 18 pear accessions were obtained by using similar methods [3]. A total of 131 pear accessions were divided into two populations (Asian population and European population), and further divided into four groups (Asian cultivated group, Asian wild group, European cultivated group and European wild group) for genetic diversity and selection signature analysis.

### Identification and classification of NBS-encoding genes

For identification of NBS-encoding genes, the Chinese white pear (*P. bretschneideri* Rehd. cv. ‘Dangshansuli’) [1] and European pear (*P. communis*.cv. ‘Bartlett’) [32] amino acid sequences were obtained from Pear Center of Nanjing Agricultural University (http://peargenome.njau.edu.cn/) and phytozome database (https://phytozome.jgi.doe.gov/), respectively. The sequence of NB-ARC domain (PF: 00931) was downloaded from Pfam database (http://pfam.xfam.org/). The software hmmer (version: 3.0) [50] was employed to search candidate NBS-encoding genes using a threshold expectation value of 1, and aiming to find the maximum number of candidate NBS-encoding genes. Also, we selected the candidate NBS-encoding genes (e-value <1E-20) as query sequence and used BLASTP [56] to search NBS-encoding genes separately in both the genomes. Subsequently, each of NBS gene in *Arabidopsis* was used as query sequence in BLASTP searching against the identified genes from above, and NCBI-Conserved Domain Database (CDD) (https://www.ncbi.nlm.nih.gov/cdd/) was used to identify the NBS domain identity in them. The same strategy was used to identify the NBS-encoding genes in *P. communis*.

The amino acid sequences of all 750 NBS-encoding genes in *P. bretschneideri* and *P. communis* genome were submitted to NCBI-CDD to identify the TIR, and LRR domain. The program COILS was used with a threshold of 0.9 [57] to specifically detect CC (coiled coils) domain in the identified genes.

### Alignment and phylogenetic tree analysis of NBS gene family

Clustalw2 [58] was employed to perform multiple alignments of the predicted amino acid sequences of gene coding region. Phylogenetic tree was constructed using the aligned amino acid sequences of the NBS proteins based on the ML (maximum likelihood) method by IQ-TREE (version 1.6.8) [59], and ultrafast bootstraps were estimated with 1000 replicates with default option.

### Cis-elements analysis of NBS-encoding genes

Promoters in the upstream 1500 bp regions of NBS-encoding genes were obtained from the pear annotation database [1] and phytozome database (https://phytozome.jgi.doe.gov/pz/portal.html). The Plant CARE (http://bioinformatics.psb.ugent.be/webtools/plantcare/html/) program was used to identify the cis-elements in promoter region of each gene. The same strategy was used to identify the cis-elements in NBS-encoding genes family of *P. communis*.

### Distribution and gene duplications of all NBS genes

Chromosomal locations of genes were displayed by using an in-house Rscript (https://www.r-project.org/). To analyze the NBS-encoding gene duplication events, the protein sequences of whole genomes were downloaded from pear genomic database and phytozome database. BLASTP was used to make an all-vs-all BLAST search (e-value of 1E-5 and top five matches). The BLAST output and the whole genome annotation file were imported to MCScanX [60] to identify homologous pairs, which were used to identify syntenic chains. The parameters were set to default as follows: Match score, 50; Overlap window, 5; Match size, 5; GAP penalty, − 1; E-value, 1E-05; Max GAPs, 25 [61]. Duplicate gene classifier [60], the core function of MCScanX was used to classify all protein-coding genes into five types of duplications including tandem, singleton, WGD/ segmental, dispersed and proximal. BLASTP output and annotation file was used as input files. The assigning procedures were as following: (1) All genes were ranked based on their order along chromosomes and were classified as ‘singletons’, and all BLASTP hit results were evaluated for further classification; (2) If the genes had BLASTP hits to other genes, genes were labeled as dispersed duplicates; (3) If the two genes in a BLASTP hit, and their difference of gene rank was lower than 20 (less than 20 other genes insertion between two genes), then they were labeled as proximal duplicates; (4) If the difference of two genes rank = 1 (no gene insertion between two genes), then they were labeled as tandem duplicates. (5) the anchor genes located in collinear blocks were labeled as WGD/segmental duplicates [60]. Finally, all duplicated genes were classified as a unique class based on the order of priority: WGD/segmental > tandem > proximal > dispersed [60]. We carried out the duplication analysis in both *P. bretschneideri* and *P. communis* using the same strategy.

### Detection of positive/purifying selection

To detect positive and purifying selection in NBS-encoding gene family, the rate of non-synonymous (Ka) and synonymous (Ks) substitutions was calculated using the Ka/Ks_Calculator2.0 [62] software with MYN method, and the ratio was obtained by dividing the Ka and Ks output from above.

### Detection of NBS-encoding genes in disease resistance QTL

QTL information was retrieved from previous studies [13, 16, 17, 22, 33–35]. The marker sequences flanking the disease-related QTL peaks were extracted (Additional file 3), and bowtie2 (version 2.3.3.1) [63] was used to map primer sequences to the reference genomes (*P. bretschneideri* and *P. communis*) with the parameter: -f -a -x and max mismatches in seed is 3. The reference genome was selected based on the QTL backgrounds from Asian or European pears. The mapping coordinates from each primer were used to define the amplification regions on pear genomes. Finally, NBS-encoding genes in these QTL flanking regions were identified for further analysis.

### SNP calling and screening SNP with significant difference

The software bwa (version 0.7.17-r1188) [64] was used to map the quality-filtered paired-end reads to the genomic sequences of 338 NBS-encoding genes. The potential PCR duplicates were removed from the alignment files using SAMtools (version 1.9) [65]. We used BCFtools (version 1.9) to perform SNP calls [66]. The low-quality SNPs were filtered out with the criteria; QUAL < 20 or DP < 4 or QUAL/DP < 2. The filtered SNP data set was used for further analysis.

After SNP calling, homozygous SNPs with missing rate < 0.1 were selected in 131 pear accessions. SNP genotypes of ‘0/1’ were excepted from the analysis. Chi-square test was performed for the SNPs at each gene locus (excepted genotype of ‘0/1’) in cultivated and wild populations, and the SNP having *p*-value < 0.01 were defined as significantly different SNP.

### Diversity of NBS-encoding genes in four groups

To measure genetic diversity of the NBS-encoding gene family in pear, nucleotide diversity (*π*) and fixation index (*F*_*ST*_) were used as population-differentiation statistics. The analysis was performed to compare the four pear groups (Asian wild, Asian cultivated, European wild, European cultivated). In brief, 338 NBS-encoding genes were combined to form a single sequence scaffold with the sorted order (Additional file 14), and SNPs were identified across them using the above defined methods. Then VCFtools (version 0.1.16) [67] was employed to further screen SNP data with the parameters: max-missing 0.50, maf 0.05. The resulting SNPs were used to calculate the *π* and *F*_*ST*_ with a 1000 bp sliding window and a step size of 500 bp across the linear sequence of all 338 NBS-encoding genes. The nucleotide diversity (*π*) was also calculated separately for the coding regions.

### Detecting signatures of selection in special genes

To identify signatures of selection, we calculated the Ka/Ks ratio of all orthologous NBS-encoding gene pairs to identify signatures of positive selection on them. Only SNPs from the coding regions were used form each reference gene. Furthermore, Ka/Ks_calculator2.0 MYN method [62] was used to calculate the Ka and Ks of each orthologous pair in wild and cultivated groups, respectively. Afterwards, sequences with Ks > 0.1 were discarded to avoid paralogs, and the average Ka, Ks and Ka/Ks ratio of each gene was calculated to identify the signatures of selection within specific group. Positive selection of genes was determined with a Ka/Ks ratio of more than one.

### Genome-wide expression analysis of the NBS-encoding genes

To investigate the expression of the NBS-encoding genes that have signatures of selection, four pear genotypes representing two cultivated and two wild accessions at three different fruit developmental stages (small, enlarged, and mature) were used. The RNA-seq data of 12 samples were obtained from a previous study [42]. To investigate the expression changes of the NBS-encoding genes after inoculation with *A. alternata*, RNA-seq data of two pear species (*P. calleryana* and *P. pyrifolia*: ‘Hongfen’ pear) were used generated from previous study [19, 20]. RPKM value was used to measure the expression level of NBS-encoding genes. The R package (https://cran.r-project.org/web/packages/pheatmap/) was used to display the expression patterns of these genes.

### Real-time PCR analysis

For gene expression validation, four pear accessions (PyW13, PyW14, PyI1 and PyL2) at enlarged stage were chosen for quantitative real time PCR (RT-qPCR) analysis. For expression validation of *Pbr025269.1* with RT-qPCR, ten Asian cultivated (PyL2, PyL7, PyL8, PyL9, PyL10, PyL11, PyI1, PyI9, PyI11, PyI14) and seven Asian wild (PyW5, PyW6, PyW7, PyW9, PyW12, PyW13, PyW14) pear accessions were chosen from a previous study [42]. Plant Total RNA Isolation Kit plus was used to extract total RNA from pear fruits at the enlarged stage, and DNase I was used to remove the genomic DNA contamination. About 1 μg of total RNA was used for cDNA synthesis by using TransScript One-Step gDNA Removal and cDNA Synthesis SuperMix (TransGen Biotech Co. Ltd.) according to the manufacturer’s protocol. Primer5.0 was used to design primer sequences for gene amplification (Additional file 15). The SYBR®Green Premix kit (TaKaRa Biotechnology, Dalian, China) was used to carry out the analysis of RT-qPCR, and the PCR mixture was composed as follows: 150 ng of cDNA, 2.5 μl of each primer (10 μM), 10 μl of 2 × SYBR Premix ExTaq TM, and 5 μl of RNase-free water. The RT-qPCR started with 10 min at 95 °C, followed by 45 cycles of 95 °C for 15 s, 60 °C for 30 s and finally 72 °C for 30s. The expression level of selected NBS-encoding genes was calculated by using 2^–ΔΔCt^ method and normalized to the *Pyrus* GAPDH gene [42].

## Supplementary Information

**Additional file 1:** **Figure S1.** A phylogenetic tree of *P. bretschneideri* NBS proteins constructed by ML (Maximum likelihood) method using IQ-TREE. Fig. S2: A phylogenetic tree of *P. communis* NBS proteins constructed by ML method using IQ-TREE. ① Two subfamilies are shown. Red represents non-TIR group and Blue represents TIR group. ② The six classes of NBS-encoding genes are marked by different colors. Green means CC-NBS-LRR type, light yellow means TIR-NBS-LRR type, yellow means NBS type, light blue means TIR-NBS type, orange means CC-NBS type and purple means NBS-LRR type. ③ Domains of NB-ARC, LRR, and TIR are displayed on the tree (CC domain was not shown). Yellow means TIR domain, red means NB-ARC domain and blue means LRR domain.

**Additional file 2.** The information of all NBS-encoding genes in *P. bretschneideri* and *P. communis*, including type, location, and length of genomic, coding and protein sequences.

**Additional file 3.** NBS-encoding genes in pear disease resistance QTL regions. A total of 18 QTL were detected from previous study [13, 16, 17, 22, 33–35]. The primers of SSR markers in QTL were used to detect the marker location on the reference genome [1, 32]. Markers upstream and downstream 500 kbp were selected to identify the presence of NBS genes related to these QTL.

**Additional file 4.** Duplication categories of NBS-encoding genes in *P. bretschneideri* and *P. communis*. Five duplication categories (Singleton, Dispersed, Proximal, Tandem and WGD) were displayed.

**Additional file 5.** Ka/Ks ratios of paralogous gene pairs in *P. bretschneideri* and *P. communis.* Paralogous gene pairs were identified using MCScanX with default parameters. ‘NA’ means no values.

**Additional file 6.** Orthologous gene pairs between *P. bretschneideri* and *P. communis*. Orthologous gene pairs were identified using MCScanX with default parameters.

**Additional file 7.** Ka/Ks ratios of orthologous gene pairs between *P. bretschneideri* and *P. communis.* ‘NA’ means no values.

**Additional file 8.** The value of nucleotide diversity (*π*) and *F*_*ST*_ within 1 kilobase sliding windows and step size of 500 bases across the whole NBS-encoding gene family from different populations.

**Additional file 9.** Significantly different SNPs between Asian (European) cultivated and wild groups. ‘0/0’ means a homozygous reference site, ‘0/1’ means a heterozygous site with two alleles (reference and variant) and ‘1/1’ means a homozygous variant site. ‘./.’ means no data. Chi-square test was used to evaluate the significantly (*p*-value < 0.01) different SNPs between cultivated and wild groups.

**Additional file 10.** The list of non-synonymous or synonymous significantly different SNPs in Asian and European populations.

**Additional file 11.** Genes with Ka/Ks ratio > 1 in Asian and European cultivated and wild pear groups.

**Additional file 12.** The RPKM values of 338 NBS-encoding genes in four Asian pear accessions at three fruit stages. Letter ‘NA’ means no data.

**Additional file 13.** The expression patterns of 338 NBS-encoding genes’ expression in *P. calleryana* and ‘Hongfen’ pear (*P. pyrifolia*). Letter ‘Treat_RPKM’ means these genes’ expression in treatment groups (inoculation of *A. alternata*). Letter ‘CK_RPKM’ means these genes’ expression in control groups. Letter ‘NA’ means no data.

**Additional file 14.** NBS-encoding genes in a sorted physical genome order. Genomic and coding sequences of 338 genes genomic were combined into a long sequence with the sorted order, respectively.

**Additional file 15.** Primer sequences of 17 genes used in RT-qPCR analysis.

## Data Availability

The *P. bretschneideri* genome datasets used in this study are available in our pear center website (http://peargenome.njau.edu.cn/). The genome and amino acid sequences of *P. communis* were downloaded from the phytozome database (https://phytozome.jgi.doe.gov/pz/portal.html). Raw WGS data of all pear accessions were downloaded from NCBI BioProject (PRJNA381668, PRJNA675194). The RNA-seq data were obtained from the NCBI SRA database (PRJNA393405, PRJNA271833, PRJNA662252). The Rscript to draw the chromosome location was submitted to (https://github.com/bioinformatic1996512/NBS).
