# Supplementary material for: Contrasting genetic variation and positive selection followed the divergence of NBS-encoding genes in Asian and European pears
Source: BMC Genomics. 2020 Nov 19;21:809. doi: 10.1186/s12864-020-07226-1 (PMC7678159; doi:10.1186/s12864-020-07226-1)
Supplement: Supplementary file 1 — Additional file 1: Figure S1. A phylogenetic tree of P. bretschneideri NBS proteins constructed by ML (Maximum likelihood) method using IQ-TREE. Fig. S2: A phylogenetic tree of P. communis NBS proteins constructed by ML method using IQ-TREE. ① Two subfamilies are shown. Red represents non-TIR group and Blue represents TIR group. ② The six classes of NBS-encoding genes are marked by different colors. Green means CC-NBS-LRR type, light yellow means TIR-NBS-LRR type, yellow means NBS type, light blue means TIR-NBS type, orange means CC-NBS type and purple means NBS-LRR type. ③ Domains of NB-ARC, LRR, and TIR are displayed on the tree (CC domain was not shown). Yellow means TIR domain, red means NB-ARC domain and blue means LRR domain. [file 12864_2020_7226_MOESM1_ESM.docx]

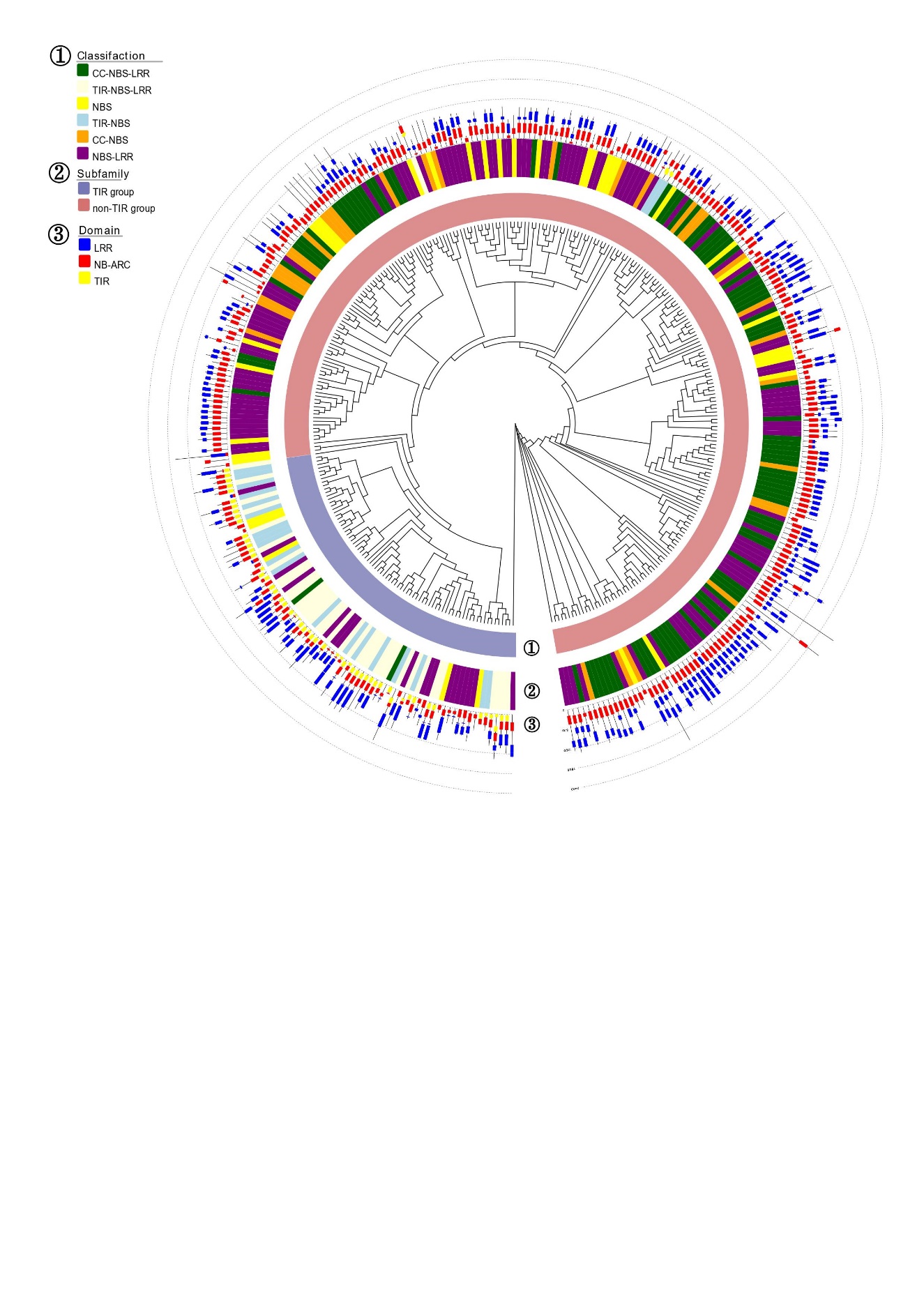


Figure S1: A phylogenetic tree of P. bretschneideri NBS proteins constructed by ML (Maximum likelihood) method using IQ-TREE. ① Two subfamilies are shown. Red represents non-TIR group and Blue represents TIR group. ② The six classes of NBS-encoding genes are marked by different colors. Green means CC-NBS-LRR type, light yellow means TIR-NBS-LRR type, yellow means NBS type, light blue means TIR-NBS type, orange means CC-NBS type and purple means NBS-LRR type. ③ Domains of NB-ARC, LRR, and TIR are displayed on the tree (CC domain was not shown). Yellow means TIR domain, red means NB-ARC domain and blue means LRR domain.


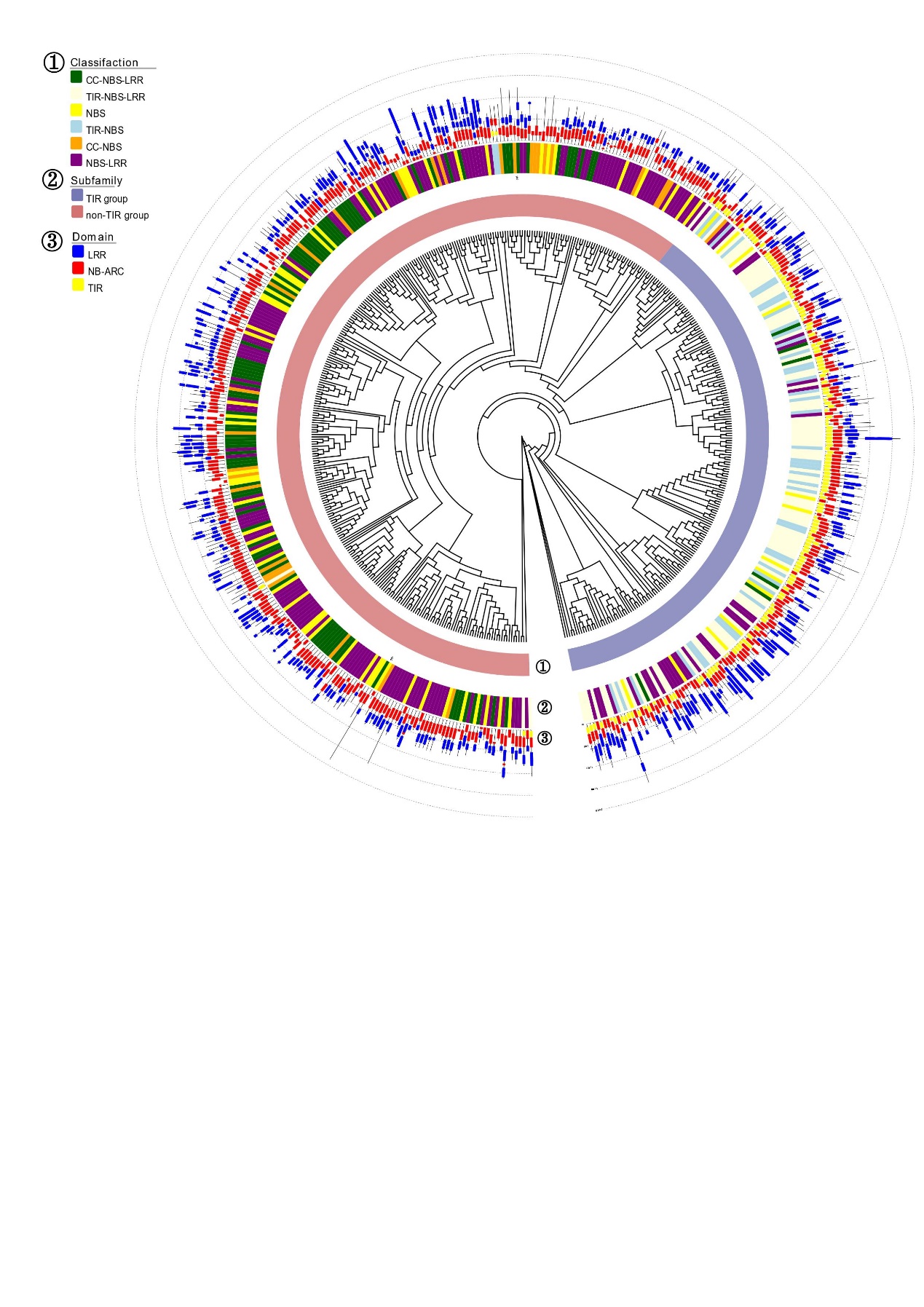


Figure S2: A phylogenetic tree of P. communis NBS proteins constructed by ML method using IQ-TREE. ① Two subfamilies are shown. Red represents non-TIR group and Blue represents TIR group. ② The six classes of NBS-encoding genes are marked by different colors. Green means CC-NBS-LRR type, light yellow means TIR-NBS-LRR type, yellow means NBS type, light blue means TIR-NBS type, orange means CC-NBS type and purple means NBS-LRR type. ③ Domains of NB-ARC, LRR, and TIR are displayed on the tree (CC domain was not shown). Yellow means TIR domain, red means NB-ARC domain and blue means LRR domain.
